# Increased brain-derived neurotrophic factor following successful psychological treatment in generalized anxiety disorder: a randomized clinical trial

**DOI:** 10.1186/s12888-026-08027-8

**Published:** 2026-04-07

**Authors:** Lucas Gandarela, Thiago P. de A. Sampaio, Lia Marçal, Emmanuel A. Burdmann, Francisco Lotufo Neto, Marcio A. Bernik

**Affiliations:** 1 Experimental Pathophysiology Program, School of Medicine, University of São Paulo, São Paulo, Brazil; 2 Department and Institute of Psychiatry, Anxiety Disorders Program, University of São Paulo, São Paulo, Brazil; 3 Department of Clinical Psychology, Institute of Psychology, University of São Paulo, São Paulo, Brazil; 4 LIM 12, Division of Nephrology, School of Medicine, University of São Paulo, São Paulo, Brazil

**Keywords:** Generalized anxiety disorder, Acceptance, Cognitive-behavior therapy, BDNF, Biomarkers

## Abstract

**Background:**

Brain-derived neurotrophic factor (BDNF) has emerged as a key regulator of neuronal plasticity in the central nervous system. Successful biological interventions upregulate the expression of BDNF in generalized anxiety disorder (GAD) patients, but no study of psychological treatments has been found. The present study aimed to evaluate changes in plasma BDNF levels following two psychological treatments with established clinical efficacy in patients with GAD. Methods: Participants were recruited for a 14-week, 10-session clinical trial of either group acceptance-based behavior therapy (ABBT) or supportive therapy (ST). Eligible patients were GAD patients aged 18–65 years. Plasma BDNF was measured in 82 patients before and after the intervention (ABBT = 41, ST = 41). Correlation with anxiety symptom severity was also assessed using the Hamilton Anxiety Scale at pre- and post-treatment. Results: BDNF increased in both groups over time (ST: difference = 2042.6, t = 4.81, *p* < 0.001 vs. ABBT: difference = 1947.8, t = 4.46, *p* < 0.001). Although anxiety symptoms decreased in both groups, no correlation between BDNF and anxiety severity was found. Conclusions: The present study suggests that a short-term trial of two clinically effective psychotherapeutic interventions was associated with increases in plasma BDNF levels in GAD patients. Given the absence of associations between BDNF changes and symptom severity, these findings should be interpreted cautiously and not as evidence of treatment-specific or mechanistic effects.

**Trial registration:**

NCT03930095||https://www.clinicaltrials.gov/ retrospectively registered on 15 April 2019.

## Background

The neurotrophin family of signaling proteins is involved in both regulating the survival, differentiation, and synapse formation of neuronal populations during development and in continuing to shape neuronal structure and function throughout the life span [1]. The brain-derived neurotrophic factor (BDNF) is a member of the neurotrophin family and has emerged as a key regulator of neuronal plasticity in the central nervous system (CNS). BDNF is distributed among brain regions particularly relevant for plasticity [2].

Psychosocial or unpredictable stress both decrease the expression of BDNF and can lead to neuronal atrophy and cell loss in brain regions associated with the pathophysiology of depression, such as the amygdala, prefrontal cortex, and hippocampus. Additionally, upregulation of BDNF expression is postulated as essential to the therapeutic actions of antidepressant treatment [3].

The implication of BDNF in the underlying neurobiology of anxiety disorders or its treatment response is not as well established as in depression. The evidence for lower BDNF levels in individuals with anxiety disorders compared to healthy controls has been reported, especially in patients with obsessive-compulsive disorder [4].

Among anxiety disorders, generalized anxiety disorder (GAD) is characterized by chronic and excessive worry and anticipatory anxiety. GAD patients have a high chance of presenting clinical depression throughout life [5], which may point to overlaps in its pathophysiological processes.

Understanding the potential role of BDNF in GAD’s pathophysiology and the neurobiological mechanisms associated with clinical improvement will lead to a better understanding of its pathophysiology and more accurate, personalized, and potentially more successful interventions.

Successful trials of biological treatments have been associated with an increase in peripheral BDNF levels in GAD patients. A 15-week placebo-controlled trial [6] of duloxetine showed an increase in plasma BDNF levels. In a smaller, non-controlled, open-label trial, serum BDNF was significantly higher after 10 daily sessions of low-frequency repetitive transcranial magnetic stimulation (rTMS) over bilateral dorsolateral prefrontal cortex [7].

Concerning psychological treatments, an increase in the serum concentration of nerve growth factor (NGF), another type of neurotrophic factor decreased by environmental stress paradigms, was observed in 22 GAD patients after 25 sessions of cognitive-behavior therapy (CBT) [8].

Among the proposed CBT modalities already proven effective in clinical trials, acceptance-based behavior therapy (ABBT) showed significant reductions in GAD symptoms compared with waitlist control [9] and comparable to applied relaxation, an older gold standard treatment for GAD [10] in randomized trials. On the other hand, the efficacy of non-directive supportive therapy has also been observed in depressed patients in comparison with other commonly used control groups such as waitlist or “treatment-as-usual” [11]. In patients with GAD, the evidence that CBT provides a better clinical response than ST is also inconclusive, with CBT providing some more favorable results in improving anxiety symptoms [12].

Finally, although psychological treatment is accepted as an efficacious alternative to biological treatments for GAD, no study to our knowledge has studied its effect on the BDNF levels of GAD patients.

The present study aimed to evaluate the effect of two psychotherapy protocols, ABBT and Supportive therapy (ST), on BDNF plasma levels of GAD patients, and whether BDNF levels would correlate with score changes in clinical anxiety symptoms measurements. We hypothesized that BDNF plasma levels of GAD patients in both psychotherapy groups would show an increase through treatment. Moreover, we hypothesized a negative correlation between BDNF plasma levels and clinical anxiety measurements.

## Methods

This study examines the effects of two psychotherapy protocols: ABBT and ST, on plasma BDNF levels in GAD patients. The results of the controlled clinical trial have already been published elsewhere [13] (Clinical Trials identifier NCT03930095).

The study was approved by the research ethics committee of the University of São Paulo Medical School, Brazil (CAPPesq), and it was conducted under the Declaration of Helsinki and Ethical Guidelines for Clinical Studies.

Data collection occurred between February and July 2016. BDNF plasma levels were measured in March 2020. All outcome measures reported here were not analyzed until data collection was complete.

### Patient population

Participants were recruited from the outpatient clinics of the Institute of Psychiatry at the University of São Paulo Medical School and through media advertisement. Individuals eligible for the study were those who (a) had a principal diagnosis of GAD as determined by the Mini-International Neuropsychiatric Interview for DSM-IV and ICD-10 (MINI) [14]; (b) were aged between 18 and 65 years; and (c) were literate. Psychiatric comorbidities such as bipolar disorder, psychotic disorder, current substance dependence, or current moderate/severe suicide risk led to exclusion. No patient could be in current psychological treatment. Concurrent pharmacotherapy was allowed if medication dosage had been stable for the three months prior to study entry.

Sample size was calculated for the primary clinical outcome in the clinical trial: the scores of the Depression Anxiety Stress Scale – 21 items [13]. A comparison of two means using R software based on Roemer and Orsillo’s results of a similar trial [9] was conducted for sample size estimation. To achieve 80% power and expecting an approximate 25% dropout rate, a total of *N* = 92 participants was needed to test the primary study aims.

### Screening and randomization

The experiment and the data collection took place at the Institute of Psychiatry of the University of São Paulo Medical School. Interested subjects underwent a preliminary screening through telephone or email. Subjects who met the inclusion criteria completed an in-person diagnostic assessment using the MINI for DSM-IV, carried out by trained psychologists or psychiatrists.

After confirming eligibility, all participants provided informed consent for the study. Each enrolled participant was randomized to a single treatment, in a 1:1 ratio, by a research assistant using a random number generator following the method of randomly permuted blocks [15]. Randomization was stratified into two blocks according to current psychotropic medication use. This same researcher was responsible for patients’ assignment to interventions.

### Interventions

Both interventions consisted of 10 two-hour group psychotherapy sessions within 14 weeks.

ABBT was modeled after the protocol developed by Roemer et al. [9] and consisted of: psychoeducation about anxiety and GAD from an acceptance-based behavioral perspective (function of emotions, the role of experiential avoidance in suffering and functional impairment), applicability of these concepts to each patient’s clinical symptoms, and a variety of mindfulness practices.

Supportive therapy was modeled after a predefined protocol following the standards for brief supportive psychotherapy [16]. In this intervention, therapists stimulated patients to participate and suggest themes for discussion to receive and offer mutual support. Besides, a nondirective psychoeducation on GAD symptoms, nosology, treatments, and epidemiology was offered. No instructions based on the acceptance-based approach of GAD, meditation, or mindfulness techniques were allowed in the ST groups. A more detailed description of the group distributions and the psychological interventions applied is published in the original clinical efficacy study [13].

### Measurement of BDNF levels

Blood samples were collected at two time points: before the first session (week 1) and after the 10th session (week 14) of ABBT or ST. At each collection, a sample of 10 mL venous blood was taken from all participants: 5 mL of whole blood in an EDTA tube and 5mL into an anticoagulant-free vacuum tube.

The samples were then centrifuged at 4000 rpm for 20 min at 4 °C, aliquoted, and frozen at -80 °C until further analysis.

Plasma levels of BDNF, expressed in pg/mL, were measured using the ChemikineTM BDNF Sandwich ELISA kit (Chemicon International, CYT306, Millipore, Billerica, MA, USA) according to the manufacturer’s instructions. All samples and standards were measured in duplicates, and the means of the duplicates were used for analyses.

### Measurement of clinical symptoms

Anxiety and depressive symptoms were measured at three time points: at baseline (week 0), before the sixth session (week 6), and after the last session (week 14).

The Brazilian Portuguese adapted version [17] of the Hamilton Anxiety Scale (HAM-A) [18] was used to measure the severity of anxiety symptoms. The interviewers were four clinical psychologists from the Anxiety Disorder Clinics staff trained in administering the scales. Raters were blinded regarding the subjects’ group allocation.

Depressive symptoms were measured using the score for depression in the Brazilian Portuguese version [19] of the Depression, Anxiety and Stress Scale – 21 items (DASS-21) [20]. DASS-21 is a self-report scale that separately measures the scores of depression, anxiety, and stress.

All self-questionnaires were completed using the software Research Electronic Data Capture (REDCap) [21], a digital platform for data collection hosted at the University of São Paulo.

### Statistical analyses

Baseline characteristics between both psychotherapy groups and completers versus non-completers were compared using Fisher’s Exact test and independent sample t-test for categorical (sex, education, marital status, psychotropic medication use, comorbidities) and continuous variables (age, HAM-A and DASS-d scores), respectively.

Mixed Effects Linear Model (MLM) was used to compare measures of BDNF, HAM-A, and DASS-d scores from baseline to post-intervention for each condition and between groups. All available data is used in this analysis, which assumes that missing data are “missing at random”. Participants were included as random effects and intercept, group, time, and covariates (baseline data) as fixed effects.

To evaluate the effects of potential confounding factors, MLMs were repeated adjusted for sex, age, baseline HAM-A score, and comorbid diagnosis of depression according to MINI. Covariates were included a priori to assess potential confounding and were not selected based on model fit criteria.

Concerning the association between clinical symptoms and BDNF, Spearman’s correlation coefficient of residual gains in percentage of each variable over time was calculated. To achieve the proposed objective, residual percentage change scores for plasma BDNF levels were first calculated between time points, following the approach described by Rankin and Tracy [22]. Residual change was defined as the proportional difference between post-treatment (T2) and baseline (T1) values relative to baseline. Specifically, residual change in plasma BDNF was calculated as: $\:({\mathrm{BDNF}}_{T2}-{\mathrm{BDNF}}_{T1})/{\mathrm{BDNF}}_{T1}$. Subsequently, Spearman’s rank correlation coefficient was used to construct the correlation matrix between changes in plasma BDNF levels and clinical variables. In the correlation analysis, missing data were excluded.

All statistical tests were conducted with statistical software SAS 9.4, and graphs were built with statistical software R 3.5.0.

An alpha value of 0.05 was established for all analyses.

## Results

**Fig. 1:**
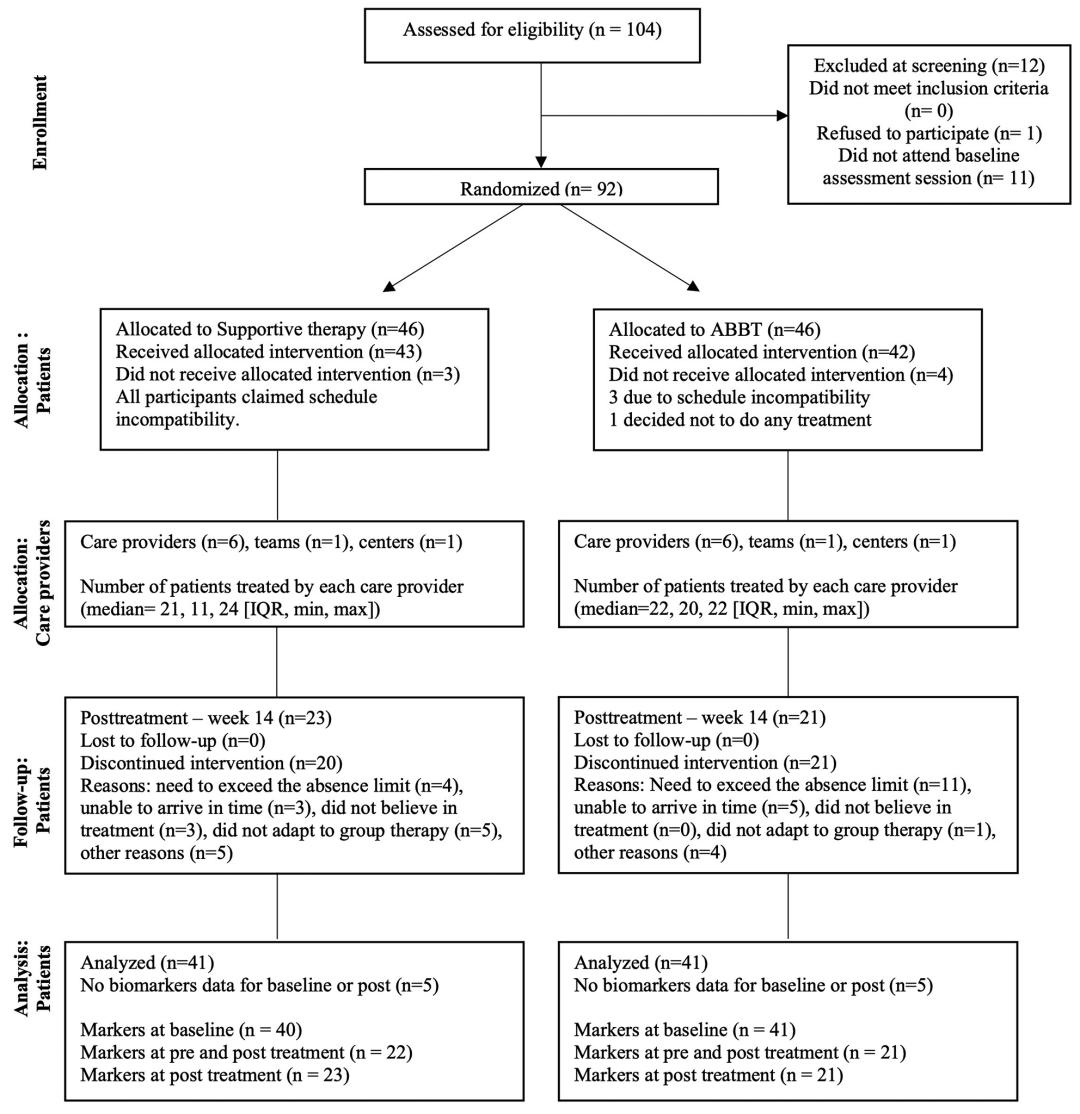
Flow chart of participants following the guidelines of the modified CONSORT flow diagram for individual randomized controlled trials of nonpharmacologic treatments [23]. ABBT, acceptance-based behavior therapy

Blood samples were obtained at least once from 82 participants out of 92 who participated in the overall randomized clinical trial (RCT) (41 of each group) (Fig. 1). 82 patients with GAD were thus included in the statistical analyses. As specified in Fig. 1, blood samples were collected from 81 participants at baseline (41 from the ABBT group and 40 from the ST group), while only 43 participants had blood samples collected at both pre- and post-treatment (21 from the ABBT group and 22 from the ST group).

### Baseline demographic and clinical characteristics

Demographic characteristics by treatment group are presented in Table 1. Participants were predominantly women (74%) with a mean age of 37 years (SD 15), who were single or divorced (77%) with completed higher education (70%). The most common psychiatric comorbidities were depression (61%) and panic disorder (39%). There were no significant group differences in terms of baseline demographic and clinical characteristics.

Regarding attrition, participants who discontinued the study did not differ from completers in terms of demographic characteristics or baseline clinical characteristics, except for diagnosis of current depression episode, which was more frequent in non-completers than completers as presented in Table 2.

**Table 1:** Baseline demographic and clinical characteristics

Outcome	Total sample (*n* = 82)	ST (*n* = 41)	ABBT (*n* = 41)	*p*-value
**Sex**				
Female(%)	61 (74%)	32 (78%)	29 (71%)	0.61
Male(%)	21 (26%)	9 (22%)	12 (29%)	
**Mean Age years (SD)**	37(15)	35(11)	38(14)	0.24
**Education**				
Elementary school(%)	2 (2%)	0 (0%)	2 (5%)	0.38
High school(%)	23 (28%)	13 (32%)	10 (24%)	
Higher Education(%)	57 (70%)	28 (68%)	29 (71%)	
**Marital status**				
Married/living together(%)	19 (23%)	11(27%)	8 (20%)	0.82
Single/divorced(%)	63 (77%)	30(73%)	33 (80%)	
**Current psychotropic use**				
Yes(%)	30 (37%)	14 (34%)	16 (39%)	0.82
No(%)	52 (63%)	27 (66%)	25 (61%)	
**Comorbidities**				
Current major depression	50 (61%)	26 (63%)	24 (59%)	0.82
Current panic disorder	32 (39%)	14 (34%)	18 (44%)	0.5
Agoraphobia	23 (28%)	15 (37%)	8 (20%)	0.14
Social Phobia	18 (22%)	10 (24%)	8 (20%)	0.8
Suicide risk	11 (13%)	6 (15%)	5 (12%)	1.00
Mean Baseline HAM-A score (SD)	27.8 (7.1)	27.8 (8.7)	27.8 (11)	1.00
Mean Baseline DASS-d score (SD)	16.4(10.4)	17.7 (11)	15 (9.7)	0.25

**Table 2:** Baseline clinical and demographic characteristics of completers and non-completers

Outcome	Completers (*n* = 43)	Non-completers (*n* = 39)	*p*-value
**Sex**			
Female(%)	29 (67%)	32 (82%)	0.21
Male(%)	14 (33%)	7 (18%)	
**Mean Age years (SD)**	36(13)	37(12)	0.81
**Education**			
Elementary school(%)	1 (2%)	1 (3%)	1.00
High school(%)	12 (28%)	11 (28%)	
Higher Education(%)	30 (70%)	27 (68%)	
**Marital status**			
Married/living together(%)	11 (26%)	8(21%)	0.61
Single/divorced(%)	32 (74%)	31(79%)	
**Current psychotropic use**			
Yes(%)	14 (33%)	16 (41%)	0.49
No(%)	29 (67%)	23 (59%)	
**Comorbidities**			
Current major depression	20 (47%)	30 (77%)	0.007*
Current panic disorder	13 (30%)	20 (51%)	0.07
Agoraphobia	11 (26%)	12 (31%)	0.63
Social Phobia	10 (23%)	7 (18%)	0.6
Suicide risk	8 (19%)	2 (5%)	0.09
Mean Baseline HAM-A score (SD)	26.7 (8.5)	28.8 (10.4)	0.38
Mean Baseline DASS-d score (SD)	15.7(10.8)	17.1 (10)	0.56

* p value < 0.05

### BDNF plasma levels over treatment

Raw values of BDNF and clinical outcomes at pre- and post-treatment are depicted in Table 3, and the results of the mixed-effects models are in Table 4.

**Fig. 2:**
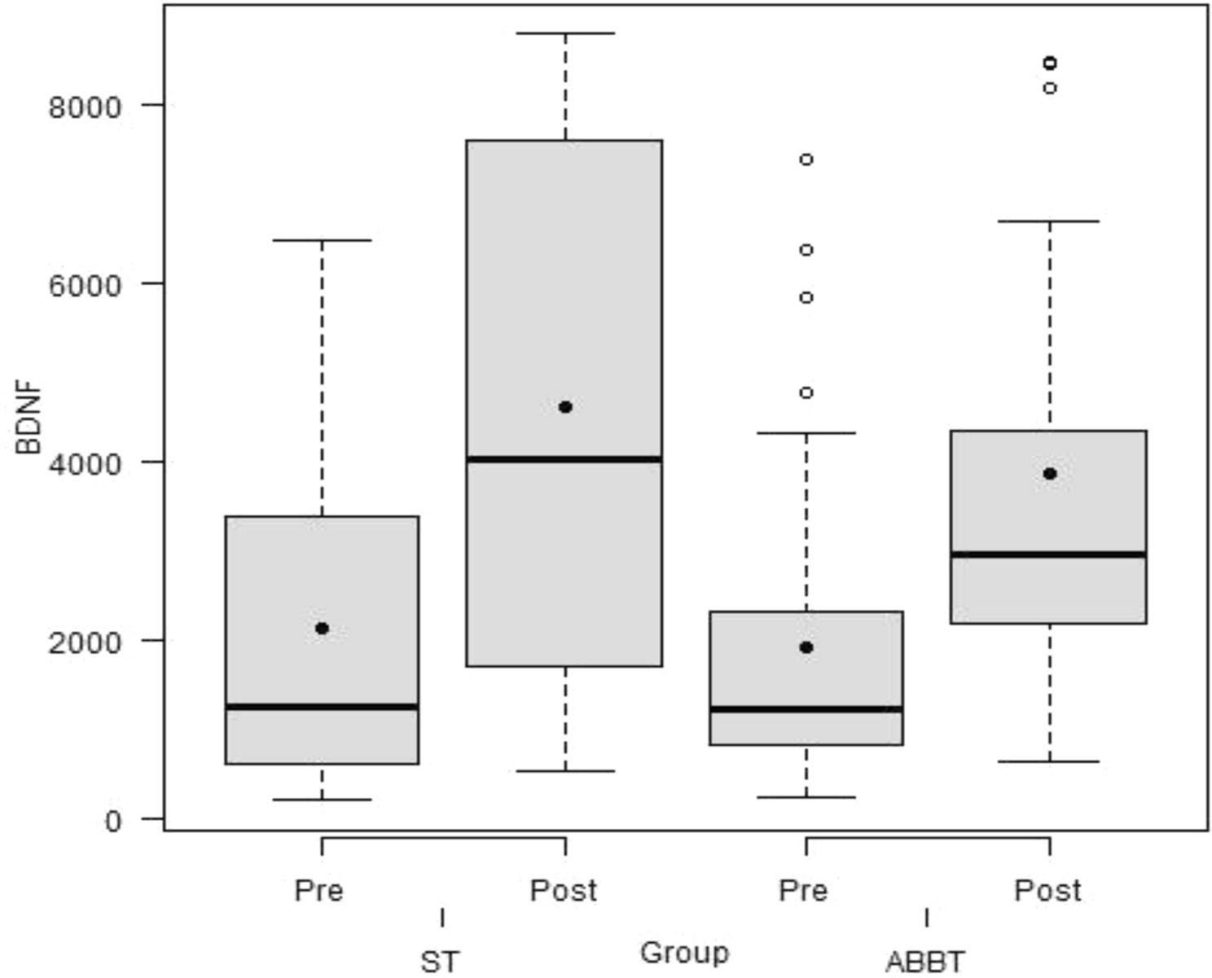
BDNF levels (pg/mL) according to intervention (ABBT=Acceptance-Based Behavior Therapy, ST= Supportive Therapy) at pre- and post-treatment. Horizontal bar = mean; black point = median; box = interquartile range (25th–75th percentiles); whiskers = values within 1.5 interquartile ranges; dots = outliers

At baseline, no significant difference in BDNF levels was observed between groups (d.f.=41, t= -0.59, *p* = 0.56). Mixed effects linear models revealed a significant time effect (F (1,41) = 42.88, *p* < 0.001) in BDNF levels, but no group *versus* time interaction was observed (F (1,41) = 0.02, *p* = 0.88). Both groups exhibited an increase in BDNF levels from pre to post treatment (ST: difference = 2042.6, CI = 1184.2 to 2900.9, t = 4.81, *p* < 0.001; ABBT: difference = 1947.8, CI = 1065.8 to 2829.8, t = 4.46, *p* < 0.001), but there was no significant difference between groups after treatment (d.f.= 41, t= -0.62, *p* = 0.54) (Fig. 2).

After adjustments to age, sex, baseline HAM-A score, and diagnosis of depression, time and group *versus* time effects were maintained. A sex effect (F (1,37) = 6.37, *p* = 0.02) was observed. No difference in BDNF levels from pre- to post-treatment was observed for male participants in the ABBT group (difference: 1093.2, CI: -457.3 to 2643.6, t = 1.43, *p* = 0.16), while BDNF showed an increase in female participants of both psychotherapy groups and in male participants of the supportive therapy group. However, no interaction group *versus* time *versus* sex (F (1,37) = 1.01, *p* = 0.4) was identified.

**Table 3:** Means (M) and standard deviations (SD) of outcomes raw values before and after treatment

Outcome	Intervention	Pretreatment M (SD)	Posttreatment M (SD)
BDNF (pg/mL)	ABBT	1906.7 (1676.3)	3864.4(2600.1)
	ST	2134.7(1931.1)	4601.8(3094.7)
HAM-A total	ABBT	27.8 (11)	11.8 (6.5)
	ST	27.8 (8.7)	16.2 (7.3)
DASS-depression	ABBT	15 (9.7)	4.9 (9.2)
	ST	17.7 (11)	6.5 (8.8)

**Table 4:** Results of the mixed-effects models examining changes across all time points

Outcome	Unadjusted			Adjusted for covariates^a^		
	df	F value	*p*	df	F value	*p*
BDNF						
Treatment	41	0.52	0.47	39	0.69	0.41
Time	41	42.88	< 0.0001*	39	32.95	< 0.0001*
Treatment x Time	41	0.02	0.88	39	0.02	0.88
HAM-A total						
Treatment	99	1.34	0.25			
Time	99	70.04	< 0.0001*			
Treatment x Time	99	1.76	0.18			
DASS-depression						
Treatment	159	0.14	0.71			
Time	159	41.02	< 0.0001*			
Treatment x Time	159	2.56	0.08			

^a^ Adjusted for sex, age, HAM-A total score, and depression diagnosis at baseline

* p value < 0.05

### Clinical symptoms over treatment

MLMs showed a significant time effect on all clinical symptom scales (Table 4): HAM-A total score (F (1,99) = 70.04, *p* < 0.0001) and DASS-depression score (F (1,159) = 41.02, *p* < 0.0001).

A significant reduction in anxiety and depressive symptoms was observed in both groups from pre- to post-treatment. However, no group x treatment effect was observed in any clinical measurement (Table 4).

### Correlation between BDNF and clinical symptoms

No significant correlation between HAM-A total, DASS-depression scores, and BDNF changes over treatment was identified (Table 5).

**Table 5:** Spearman correlation coefficient of residual gains (%) over time between BDNF plasma levels and clinical symptoms

		HAM-A total score	DASS-depression
BDNF	Correlation	-0.10	-0.13
	n	44	41
	p-value	0.51	0.44

## Discussion

This study analyzed the effect of two clinically effective 14-week psychotherapy protocols on BDNF levels in GAD patients. An increase in plasma BDNF levels was observed after both ABBT and supportive therapy groups, with no difference between them. However, no correlation between anxiety and depressive scores changes and BDNF change over treatment was observed in this trial.

The increase in BDNF levels after effective psychological treatments is consistent with the results of other studies that investigated the effect of validated treatments for GAD on neurotrophins. An increase in the concentration of BDNF was demonstrated in GAD patients after a 15-week placebo-controlled trial of duloxetine [6], and an increase in nerve growth factor levels was also observed after successful CBT [8] in GAD patients.

As GAD patients in both psychotherapy protocols exhibited significant decreases in anxiety and depressive symptoms, an increase in BDNF levels of both groups was not unexpected.

However, in our study, we could not find a significant correlation between BDNF and anxiety severity changes from pre- to post-treatment. This result is also consistent with previous studies, which did not observe a precise association between anxiety and depression symptoms rating scale score changes and changes in BDNF concentrations in anxiety disorder patients. Accordingly, Ball et al. [6] did not find an association between baseline anxiety severity levels and the concentration of BDNF in GAD patients. In addition, the mean increase in BDNF levels for the responder group did not differ from the non-responder group. Kobayashi et al. [24] also did not find a correlation between BDNF serum concentration and anxiety severity in panic disorder patients, despite the difference in BDNF levels between responders and non-responders to CBT.

A potential confounding factor could be that the increase in BDNF was due to changes in depressive symptoms rather than anxiety symptoms, given the usual comorbidity between both diagnoses [25]. To address that possibility, the correlation between depressive symptoms severity and BDNF plasma levels was calculated, but there was also no significant correlation between those variables.

The absence of correlation between anxiety and depression symptoms and BDNF levels concurrent with the increase of BDNF levels over both psychological treatments may be better explained by an overall improvement in psychological distress or psychopathological symptoms [26] that could have been provided by nonspecific factors of both protocols such as social support and learning of coping strategies.

Taken together, the absence of differential effects between ABBT and supportive therapy suggests that observed changes in plasma BDNF are unlikely to reflect treatment-specific mechanisms tied to particular therapeutic techniques. Rather, these findings are more consistent with the involvement of nonspecific or shared mechanisms of psychotherapeutic change. From this perspective, BDNF may be better conceptualized as a peripheral biological marker associated with broader psychosocial processes, such as therapeutic engagement, emotional regulation, or stress reduction, which are common across different therapeutic approaches.

In addition, in the absence of a waitlist or non-treated control group, increases in BDNF cannot be unequivocally attributed to treatment effects alone and may also reflect spontaneous symptom fluctuation, regression to the mean, or nonspecific factors related to study participation.

Although studies in animal models have reported correlations between peripheral and central nervous system BDNF levels [27], several methodological limitations related to peripheral BDNF assessment should be acknowledged. In the present study, BDNF was measured in plasma rather than serum, following prior studies examining the effects of psychotherapy and pharmacological treatments on BDNF [6, 28]. While plasma BDNF may be less influenced by platelet degranulation during coagulation, it remains a peripheral measure subject to biological variability and potential confounding. Several factors known to influence peripheral BDNF levels such as weight, physical activity, smoking, hormonal status were not systematically assessed; therefore, residual confounding cannot be excluded. Accordingly, plasma BDNF findings should be interpreted cautiously and not overinterpreted as direct indicators of central neuroplastic processes.

Finally, this is the first RCT that investigated the effect of effective psychological treatments on BDNF peripheral levels in GAD patients. Most studies on the impact of psychotherapy on BDNF levels were not randomized and have examined other psychiatric disorders such as depression, post-traumatic stress disorder, or bulimia [28].

### Limitations

There were methodological limitations in the present study. Dropout rates were higher than expected (54% in the experimental group and 50% in the control group). Attrition and missing biological samples may have limited statistical power and increased the risk of type II error. In this context, secondary and subgroup analyses, such as sex-related effects, were exploratory in nature and should be interpreted with caution. Baseline comparisons indicated that non-completers had a higher rate of diagnosis of depression than completers, suggesting selective attrition and the potential underrepresentation of individuals with greater stability of depressive symptoms in the final analytical sample. These factors may have affected internal validity and limit the generalizability of our results.

Although it was not directly assessed, some possible explanations for the dropout rate in our study can be speculated: possible comorbid blood-injection-injury phobia in GAD patients, the number of patients who did not show up for the first session (when baseline blood samples were collected), the strict limit on absences allowed in the clinical protocol which lead to patient exclusion from the protocol, and insufficient emphasis on the importance of the blood collection for the participants. Another limitation of the present study findings is unmeasured or confounding variables that may have influenced BDNF concentrations: time of collection [29], smoking [30], physical activity [31], and other medical conditions (e.g., polycystic ovary syndrome [32]).

## Conclusions

In conclusion, the present study suggests that short trials of both acceptance-based behavior therapy and supportive therapy were associated with an increase in BDNF plasma levels. However, these changes were not correlated with improvements in anxiety or depressive symptoms, underscoring the need for caution in interpreting their clinical significance. Future studies are needed to further examine the relationship between symptom change and BDNF levels in patients with generalized anxiety disorder undergoing psychological treatment, including comparisons with non-treated control group and with other interventions previously associated with BDNF changes, such as pharmacological treatment with antidepressants.

## Data Availability

The datasets used and/or analysed during the current study are available from the corresponding author on reasonable request.

## Abbreviations

BDNF: Brain-derived neurotrophic factor
GAD: Generalized anxiety disorder
ABBT: Acceptance-based behavior therapy
ST: Supportive therapy
CNS: Central nervous system
rTMS: repetitive transcranial magnetic stimulation
NGF: Nerve growth factor
CBT: Cognitive-behavior therapy
MINI: Mini-International Neuropsychiatric Interview
HAM-A: Hamilton Anxiety Scale
DASS-21: Depression, Anxiety and Stress Scale – 21 items
REDCap: Research Electronic Data Capture
MLM: Mixed Effects Linear Model
RCT: Randomized clinical trial
